# Mesenchymal stem cells and their microenvironment

**DOI:** 10.1186/s13287-022-02985-y

**Published:** 2022-08-20

**Authors:** Jiaxi Liu, Jinfang Gao, Zixie Liang, Chong Gao, Qing Niu, Fengping Wu, Liyun Zhang

**Affiliations:** 1 Third Hospital of Shanxi Medical University, Shanxi Bethune Hospital, Shanxi Academy of Medical Sciences, Tongji Shanxi Hospital, Taiyuan, 030032 China; 2 Department of Biomedical Engineering, Duke University Pratt School of Engineering, Durham, NC 27708 USA; 3 Department of Pathology, Harvard Medical School, Brigham and Women’s Hospital, Boston, MA USA

**Keywords:** Mesenchymal stem cells, Microenvironment, Immunomodulation, Tissue regeneration

## Abstract

Mesenchymal stem cells (MSCs), coming from a wide range of sources, have multi-directional differentiation ability. MSCs play vital roles in immunomodulation, hematopoiesis and tissue repair. The microenvironment of cells often refers to the intercellular matrix, other cells, cytokines and humoral components. It is also the place for cells’ interaction. The stability of the microenvironment is pivotal for maintaining cell proliferation, differentiation, metabolism and functional activities. Abnormal changes in microenvironment components can interfere cell functions. In some diseases, MSCs can interact with the microenvironment and accelerate disease progression. This review will discuss the characteristics of MSCs and their microenvironment, as well as the interaction between MSCs and microenvironment in disease.

## Introduction

Mesenchymal stem cells (MSCs) can be isolated from a variety of tissues and differentiate into mesodermal lineage cells(such as adipocytes, osteocytes and chondrocytes) and other ectodermal lineage cells(neuronal and neuroglial cells) [1]. MSCs express surface markers CD73, CD90 and CD105, but do not express hematopoietic markers CD45, CD34, CD14 and CD79 [2]. MSCs that come from different tissues have many common surface markers (Table 1).They are widely used in cell therapy, tissue engineering and regenerative medicine for their self-renewal, pluripotency and immunomodulatory properties [3, 4].

**Table 1:** Surface markers expressed by MSCs from different sources

Sources	Surface markers	Application prospect
Adipose	CD13, CD90, CD105, STRO-1 [5]	Osteoarthritis, Multiple Sclerosis[6]
Bone marrow	CD90, CD105, CD146, CD271[7]	Stroke, Parkinson’s disease, Alzheimer’s disease[8]
Synovial membrane	CD44, CD73, CD90, CD105[9]	Rheumatoid arthritis, cartilage repair[10]
Perivascular	CD90, CD105, CD248, CD271[11]	Rheumatoid arthritis, bone regeneration[11, 12]
Umbilical cord blood	CD44, CD73, CD90, CD105[13]	Ameliorating psoriasis-like skin lesion[14]
Umbilical cord tissue	CD44, CD73, CD90, CD105[15]	Corneal epithelial repair[15]
Placenta	CD29, CD73, CD90, CD105[16]	Attenuating spinal cord injury in mice [16]
Menstrual blood	CD29, CD73, CD90, CD105[17]	Treating critically ill COVID-19 patients [17]
Dental pulp	CD73, CD90, CD105[18]	Autism spectrum disorder,metatropic dysplasia [19]

**Fig. 1:**
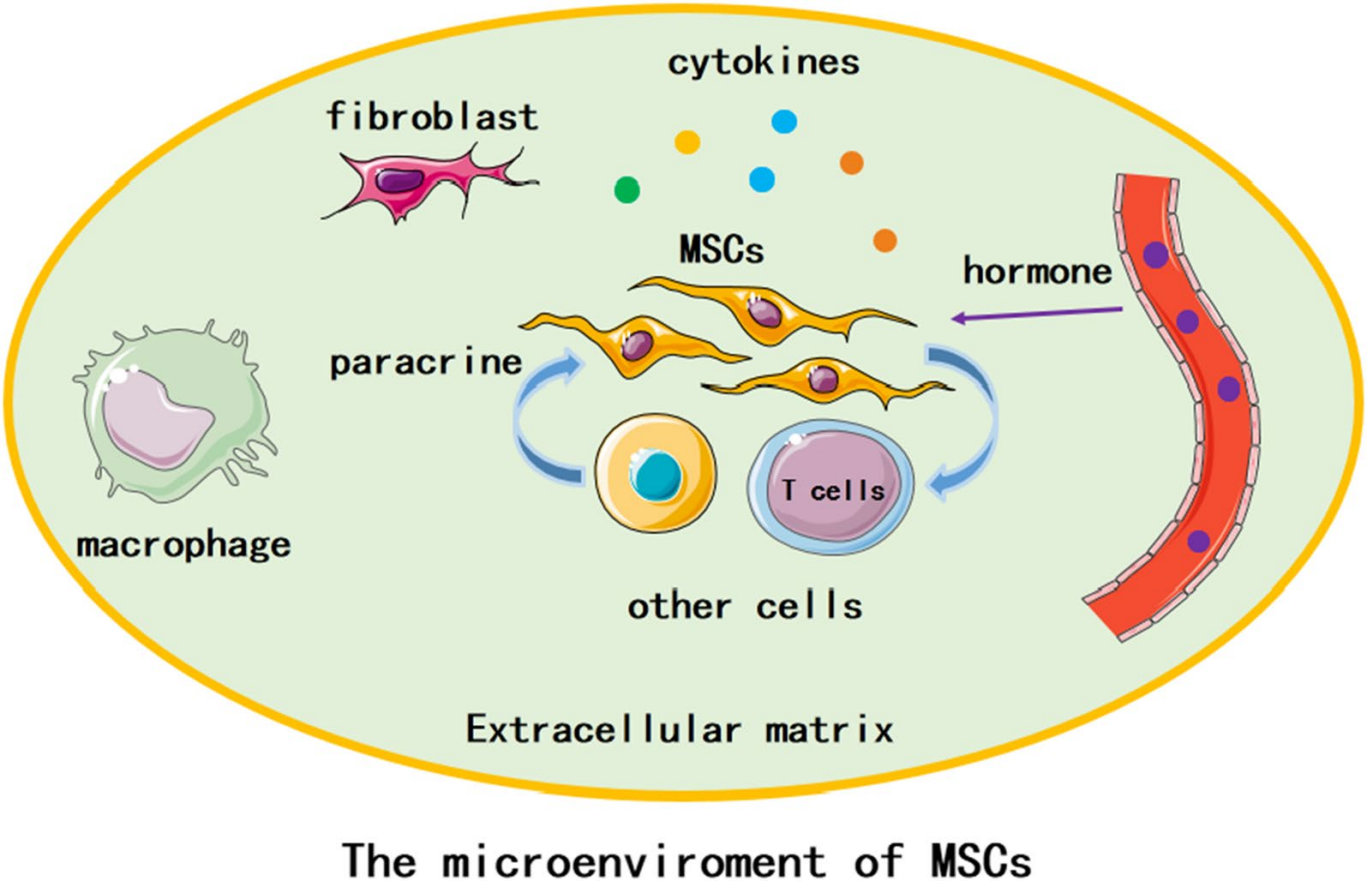
The microenvironment of MSCs. Components of MSCs microenvironment. Green background means extracellular matrix (ECM). MSCs, macrophages, fibroblasts and other cells locate in the ECM. MSCs and other cells can interact through cytokines. Hormone in blood vessel enters the microenvironment and affects MSCs

Cell microenvironment consists of components which directly influence conditions around one cell or group of cells. It has direct or indirect impact on cell behavior through biophysical, biochemical, or other ways. Generally, cell microenvironment contains extracellular matrix (ECM), homotypic or heterotypic cells surrounding the single cell, cytokines, hormones, and mechanical forces from the movement of the organism or physiological fluids (Fig. 1) [20]. For the microenvironment of MSCs, it interacts with MSCs to regulate MSCs proliferation and differentiation [21].MSCs expand in the microenvironment and receive growth signals that determine their cell fate. These signals include the interaction among cells, cells and matrix, and the transcriptional program of activating and/or inhibiting MSCs genes [22].

## Regulation of MSCs on the microenvironment

### Immunomodulation

#### MSCs effects on Macrophage

MSCs regulate macrophages mainly through the secretion of soluble factors [23]. For example, MSCs can promote the differentiation of macrophages from proinflammatory phenotype M1 to anti-inflammatory phenotype M2 by secreting prostaglandin E2(PGE2),TGF-β and CCL2 [24–26]. Moreover, bone marrow MSCs affect macrophages through extracellular vesicles. The extracellular vesicles can reduce colitis in mice by inducing colonic macrophage polarization in the immunosuppressive M2 phenotype [23].

In the liver injury model, MSCs reduce hepatocyte damage through increasing the activity of the Hippo pathway, which can activate NLRP3 and regulate XBP1-mediated NLRP3, leading to the differentiation of macrophages from M1 to M2 phenotype [27]. Additionally, murine adipose-derived mesenchymal stem cells (AD-MSCs) were found to upregulate the ratio of M2-like cells by increasing the secretion of IL-10 in mice [28].

#### MSCs effects on neutrophils

Neutrophil phagocytic activity can be increased by the secretion of IL-17 in MSCs. Bone marrow MSCs reduce the production of reactive oxygen species (ROS) in activated neutrophils, and umbilical tissue-derived MSCs reduce inflammatory activity in neutrophils [29].Furthermore, MSCs can inhibit neutrophils infiltration into sites of inflammation in a TSG6-dependent manner [30].

Activated human umbilical cord blood-derived MSCs can secrete IL-8 and macrophage migration inhibitory factors to recruit neutrophils to engulf the MSCs. Those gathered neutrophils contact or exert paracrine effects on other neutrophils, increasing the function and viability [31].

When bacterial infection happens, neutrophils go through respiratory burst. MSCs can suppress the respiratory burst and delay the apoptosis of resting neutrophils mediated by IL-6,making more neutrophils survive [32].

#### MSCs effects on T cells

MSCs can maintain macrophages and dendritic cells in an immature or anti-inflammatory state, which prevents the activation of effector T cells and promotes the formation of regulatory T(Treg) cells [33, 34]. MSCs can also secret immunomodulation molecules, such as TGF-β1 [35], leukemia inhibitory factor [36] and indoleamine2,3-dioxygenase(IDO) [23]. When CD4^+^ helper T cells and cytotoxic CD8^+^ T lymphocytes produce interferon-γ(IFN-γ),bone marrow MSCs obtain T cell inhibitory properties [37].

Human umbilical cord derived MSCs inhibit T lymphocyte proliferation and downregulate RORγt mRNA and protein expression. Those MSCs also reduce the ratio of Th17 cells and increase the ratio of regulatory T cells(Treg cells) in the spleen. In addition, they can also downregulate RORγt and Foxp3 expression in joints. Finally, these regulations lead to improvement of arthritis, delay of radiological progress, and inhibition of synovial hyperplasia in CIA rats [38, 39].

#### MSCs effects on B cells

As for B cells, adipose tissue derived MSCs can inhibit plasma cell formation and promote the production of regulatory B cells (Breg cells) [40]. Breg cells that produce IL-10 have been proved to transform effector CD4^+^ T cells into Foxp3^+^ Treg [41]. In the presence of T cells, MSCs can still inhibit the proliferation of B cells [42].

MSCs suppress B cells proliferation through inducing G0/G1 cell cycle arrest and secreting Blimp-1, a soluble factor for antigen production. The intercellular communication also plays a vital role in MSC-based B cells immunosuppression, which is medicated through PD-1 [43].

### Hematopoietic support

In bone marrow, hematopoietic stem cells (HSCs) and MSCs are closely related. Human HSCs surface markers include CD34^+^, CD90^+^ and CD105^+^. They can differentiate into myeloid cells, lymphocytes, erythrocytes or megakaryocytes [44].

The bone marrow microenvironment protects hematopoietic characteristics, such as survival, self-renewal and differentiation of HSCs, and maintains the normal function of the blood system under normal and stress conditions [45].

Bone marrow MSCs regulate their microenvironment through a variety of pathways. Notch ligands in MSCs play an important role in the survival and proliferation of hematopoietic stem cells through Wnt pathway[46].In the early stage of primary bone marrow fibrosis, the original hematopoietic support and self-characteristic related gene expression of MSCs began to decrease significantly. Extracellular matrix secretion, ossification and myofibroblast related gene expression began to increase significantly. MSCs secrete extracellular matrix(including collagen, matrix bodies, etc.) and differentiate into fibroblasts through TGF-β signal related pathways to drive bone marrow fibrosis [47].

Dysfunction of bone marrow MSCs is associated with the impaired bone marrow microenvironment that promotes leukemia development. Bone marrow MSCs treatment can reduce tumor burden and prolong survival in leukemia-bearing mice. Donor bone marrow MSCs treatment can reprogram host macrophages into arginase 1 positive phenotype with tissue repair features. Then, transfusion of MSC-reprogrammed macrophages recapitulate the therapeutic effects of MSCs. Therefore, donor MSCs reprogram host macrophages to restore the bone marrow microenvironment and inhibit leukemia development [48].

### Tissue repair and regeneration

MSCs can secrete a variety of factors supporting cell survival, including growth factors, cytokines and extracellular matrix. Adipose-derived MSCs can act on vascular endothelial cells, promote angiogenesis and maturation [49]. In addition, in many injury models such as myocardial ischemia-reperfusion and stroke, adipose derived stem cells exosomes can promote angiogenesis and reduce tissue injury [50]. Bone marrow MSCs can improve the joint inflammation of CIA (collagen induced arthritis) rats, inhibit bone damage and repair cartilage damage through regulating the proportion and function of T and B cells, bone metabolic factors and self-differentiation into chondrocytes [51].

The liver microenvironment contain cells, extracellular matrix, cytokines, and nutrition, and all of them are in homeostasis. It is a multidirectional interaction complex that plays a vital role in the maintenance of normal functions [52]. MSCs affect the liver microenvironment through a paracrine effect, modulating immune responses and homing into the injury site, which directly/indirectly build a regenerative microenvironment and repair injured tissues. MSCs can regulate the proliferation of hepatic stellate cells and the modulation of ECM, inducing apoptosis of hepatic stellate cells by secreting HGF, IL-10, and TNF-α [43]. Bone marrow MSCs have been proven to restore the albumin level and suppress the liver fibrosis in rodents [53]. Human amnion- and chorion-derived MSCs can stimulate the proliferation of hepatocytes and induce liver regeneration by secreting several active cytokines, such as HGF,EGF, and NGF, even during fulminant failure [54].

In liver injury, human umbilical cord MSCs reduce hepatocyte injury by reducing the level of inflammation and participate in the repair of hepatocyte injury after hepatic ischemia-reperfusion. The MSCs can inhibit the chemotactic recruitment of neutrophils in the inflammatory environment. MSCs may alleviate hepatic ischemia-reperfusion injury by reducing the recruitment of neutrophils in the liver [55].

## Regulation of the microenvironment on MSCs

The microenvironment is vital for cell activity and function. Different microenvironments have different effects on the biological function of MSCs. The differentiation potential of MSCs highly depends on microenvironmental soluble factors, including cytokines(IL-6 and TNF-α), hormones(estrogens, parathyroid hormone and growth hormone) and growth factors(TGF-β,IGF-1,VEGF and FGF) [22].

### Microenvironment and MSCs differentiation

#### Osteogenic differentiation

In physiological conditions, the microenvironment can support MSCs and determine their fate. In the oral tissue of mice, Runx2^+^ microenvironment cells locate at the lateral edge of the cervical loop. These cells maintain the stability of incisor mesenchymal tissue through IGF-2 signal. Runx2 genes can encode transcription factors, which are very important in the early development of bone and incisor. It was found that Runx2 is expressed in Gli1^+^ cell subsets in the proximal region of incisors, and the deletion of Runx2 would damage the growth rate of incisors in mice. When mouse dental pulp MSCs differentiate into odontoblasts and dental pulp cells, the location of Runx2^+^ microenvironment cells did not change [56].

IGF1,one of the most abundant growth factors deposited in the bone matrix, can enhance osteogenic differentiation of bone marrow MSCs via the mTOR pathway. Moreover, estrogens can bind their α and/or β receptors and induce bone marrow MSCs osteogenic differentiation through the activation of p38 MAPKs/NF-κB and BMPs/WNT/β-catenin signaling pathways [22].

#### chondrogenic differentiation

Differentiation of MSCs into chondrocytes requires a various promoters and inhibitors. The microenvironment contains soluble cytokines, nearby cells, surrounding matrix, and physical stimuli, all of which play an vital role in determining the cellular fates and chondrogenic differentiation of MSCs [57]. For example, minimum level of Wnt signaling activity is necessary to allow the chondrogenesis of MSCs. Mild activation of the pathway is needed for the chondrogenesis, as overexpression of Wnt signaling causes harmful effects on chondrogenic differentiation [58]. Furthermore, TGFβ,the most abundant growth factors in bone marrow microenvironment, originates mainly from bone matrix degradation and activated T cells. It promotes bone marrow MSCs chondrogenesis by stabilizing SOX9 via the SMAD or the p38 pathways. IL-6 can impair MSCs ability to generate chondrocytes and keep them in an undifferentiated state by activating ERK1/2 [22].

### microenvironment and MSCs aging

Aging microenvironment has adverse effects MSCs function. It affect MSCs through senescence-associated secretory phenotype(SASP),extracellular vesicles derived from senescent cells, and cell–cell contact [59].

Senescent cells can secrete bioactive factors. These factors include pro-inflammatory cytokines,chemokines, growth modulators, proteases, and the factors are termed SASP [60]. As for SASP, it contains many aging-related elements. These elements affect the function of MSCs, including proliferation, clonal formation, differentiation, immune characteristics, telomerase activity, cell migration, and adhesion. For example, the SASP can create a chronic inflammatory microenvironment that is mainly mediated by NF-κB signaling and thus leading to MSCs dysfunction and aberrant remodeling [61]. As a kind of chemokines, aging-associated insulin-like growth factor-binding protein 4 and 7 could directly induce a senescent phenotype in MSCs [62].

A senescence-associated increase in extracellular vesicles secretion can induce senescence of adjacent cells [59]. With aging, senescent cells-derived microRNA-183-5p induces MSCs senescence [63]. Very long-chain C24: 1 ceramide is increased in extracellular vesicles with aging, and those visicles can induce senescence of MSCs [64].

### hypoxia microenvironment and MSC

Hypoxia microenvironment plays a vital role in keeping the phenotype of undifferentiated MSCs. It helps MSCs stay in a quiescent status and have a necessary self-renewal rate. Furthermore, the hypoxia inducible factor(HIF) act as a molecular regulator for hypoxia microenvironment to control differentiation and survival of MSCs [65].

Bone marrow MSCs are often in a hypoxic microenvironment, which plays an important role in inducing osteogenic differentiation and increasing chemotaxis migration [66, 67]. As for the umbilical cord MSCs, hypoxia microenvironment promotes their proliferation through HIF-1, and the oxygen consumption rate of bone marrow MSCs is reduced about 3 times. The MSCs differentiate less than the MSCs under normoxic conditions. In the hypoxia microenvironment, MSCs have larger and less complex nuclei, richer nucleoli and higher nuclear/cytoplasmic index, while the cell sizes are similar to MSCs under normoxic conditions [68].

Hypoxic microenvironment can also regulate the immune response of human gingiva-derived MSCs. Fas-FasL pathway mediates apoptosis of many cell types.IL-10 turns cytokines into anti-inflammatory mediators. In human gingival MSCs, 12–24 h 2% hypoxia treatment increase the production of IL-10 and the expression of FasL. The higher expression level of FasL enhances the inhibitory effect on the proliferation of peripheral blood monocytes. Hypoxic preconditioning is an ideal method to optimize the regeneration and therapeutic potential of MSCs. However, if the oxygen concentration is too low or the hypoxia time is too long, the function of bone marrow MSCs may be lost [69]. Moreover, hypoxic cultured gingiva-derived MSCs have increased expression of stemness-related gene NANOG and neurotrophic factors VEGF and IGF1 [70].

For adipose tissue derived MSCs, hypoxia enhances their regenerative potential and does not hinder their immunomodulatory effects [71]. Additionally, 5% O_2_ significantly enhance the tenogenic differentiation of adipose tissue derived MSCs, and activated their VEGF expression [72]. Furthermore, there is an increased expression of HIF1α, which increase COL2A1 and aggrecan expression [73].

Different oxygen concentration have different impact on adipose tissue derived MSCs.2% O_2_ increases their proliferation, viability, soluble factors secretion with a low risk of tumor genesis and genetic instability. Trilineage differentiation potential of adipose tissue derived MSCs was found to be varied under different concentrations of oxygen. 1% O_2_ and 1.5% O_2_ was found to maintain adipogenic, osteogenic and chondrogenic differentiation of adipose tissue derived MSCs. 2% O_2_ and 5% O_2_ was found to increase their chondrogenesis while reduce their adipogenesis and osteogenesis[74].

## Interaction between MSCs and microenvironment in diseases

Different pathological microenvironments have different effects on MSCs, such as growth, proliferation, migration, apoptosis and differentiation, which also depends on the type and severity of the disease [75]. On the other hand, MSCs can also affect the pathological microenvironments. Some effects may aggravate the disease, while some effects alleviate the disease.

### Interaction between microenvironment and MSCs in tumor

Tumor microenvironment is a complex entity. Different tumor types have different composition of the tumor microenvironment. However, there are some hallmark features, including immune cells, stromal cells(including MSCs), blood vessels, extracellular matrix, adipose cells, and soluble factors. The tumor microenvironment is not just a bystander. It is an active promoter of cancer progression” [76].

MSCs may play a vital role in the generation of most stromal components of the tumor microenvironment. Cancer-associated MSCs differentiate into tumor supporting carcinoma-associated fibroblasts and adipocytes in the presence of tumor cells. Both resident and distally recruited MSCs have acquired a carcinoma-associated fibroblasts-like phenotype within the tumor microenvironment niche [77].

Within the tumor microenvironment, cancer-associated MSCs demonstrate a greater ability to differentiate into carcinoma-associated fibroblasts versus normal MSCs [78]. The pro-tumorigenic functions of carcinoma-associated fibroblasts contain increased tumor cell invasion, enhanced epithelial-mesenchymal transition through Hedgehog signaling, promotion of migration and metastasis, and increased chemotherapeutic resistance [79]. Adipocytes in tumor microenvironment can generate growth factors, hormones, cytokines, and adipokines.Increased insulin-like growth factor binding protein-2 expression and secretion in carcinoma-associated adipocytes enhances migration and invasion in breast cancer models [80].

Tumor cells can secrete soluble factors that promote MSCs to migrate to the site of tumor. MSCs that gather near tumor cells differentiate into more matured MSCs. Then the phenotypes and gene expression of those MSCs change. After MSCs are recruited to the tumor microenvironment, they can enhance the metastatic potential of tumor cells. Furthermore, those MSCs promote the formation of neovascularization at the tumor site by secreting VEGF. In addition, MSCs also enhance the invasion and migration of prostate cancer cells by increasing the expression of MMP2 and MMP9. The discovery of MSCs at the tumor growth site often indicates the further deterioration of the disease. Therefore, MSCs can be used as a marker of tumor progression [81].

### Interaction between inflammatory microenvironment and MSCs in RA

The synovium is the crucial location where pathogenic events develop in rheumatoid arthritis(RA). The stromal cells(including MSCs), extracellular matrix molecules, immune cells, and other tissue resident cells comprise synovial tissue microenvironment. The stromal cells contribute to the tissue architecture and regulate the tissue function [82]. In the inflamed tissues, many inflammation-related substances, such as inflammatory cells, pro-inflammatory enzymes, and inflammatory mediators form inflammatory microenvironment [83]. Abnormalities in the MSCs microenvironment, such as chronic inflammation, biotoxins, somatic stress and chemical harmful substances, will have negative effects on MSCs [84].

MSCs extracted from RA patients have significant immunosuppressive effect in vitro, such as inhibition of T lymphocytes. However, the articular microenvironment of RA reduces the efficiency of MSCs in regulating immune responses [85]. Synovial MSCs in the inflammatory microenvironment of RA can induce the conversion of macrophages to pro-inflammatory phenotype through TLR2 and TLR4. MSCs can inhibit the differentiation of monocytes into dendritic cells through various mechanisms such as IL-6, TSG-6, COX-2/PGE2 [86].

T lymphocytes play a central role in the inflammatory response related to RA. T lymphocytes widely affect the functions of MSCs. Cytokines secreted by T lymphocytes, such as IFN-γ and TNF-α,can promote the migration ability of MSCs and can be upregulated [87].

De Bari inferred that the diseased inflammatory microenvironment in RA has changed the immunomodulation of membrane synovial MSCs. These MSCs may turn into harmful cells and even lead to pannus formation [88].

### Interaction between microenvironment and MSCs in intervertebral disc degeneration

Intervertebral disc degeneration is a chronic progressive process associated with exhaustion of the resident cell population, degradation of the extracellular matrix, tissue inflammation and dehydration of the nucleus pulposus [89]. The degenerative intervertebral disc microenvironment is characterized by hypoxia, low glucose levels, acidic pH, hyperosmolarity, inflammation, and mechanical loading [90]. MSCs can differentiate into mature cells, secrete growth factors and cytokines to support resident cell activity and induce endogenous repair of the degenerated intervertebral disc [91].

Changes in the physical and chemical microenvironment of intervertebral disc (IVD) (i.e. hypoxia, reduced nutrition and acidic condition) may lead to intervertebral disc degeneration. In the degenerative disc microenvironment, hypoxia activates HIF-1α/ Yap signaling pathway, protecting mouse bone marrow mesenchymal stem cells from mechanical stress-mediated apoptosis [92].

Grafted MSCs are capable of restoring intervertebral disc degeneration back to the normal disc via stimulation of the generation of extracellular matrix proteins such as aggrecan, proteoglycan, and collagen type-II, which constitute nucleopulpocytes (chondrocyte-like round cells usually located within the nucleus pulposus) [91, 93]. MSCs are capable of differentiating into nucleopulpocytes-like phenotypes [94].

The differentiation of MSCs into a nucleopulpocytes-like phenotype was augmented by growth factors like TGF-β, PDGF, IGF-1, GDF-5 and bFGF. These factors are secreted by intervertebral disc resident cells [90, 94]. One the other hand, MSCs were capable of expressing IGF-1 and BMP-7, which protected nucleopulpocytes against apoptosis [95]. Introduction of nucleus pulposus MSCs to the hypoosmotic microenvironment of mild intervertebral disc degeneration revealed an upsurge of nucleus pulposus MSCs proliferation as well as chondrogenic potential [96]

### Interaction between microenvironment and MSCs in pulmonary fibrosis

During pulmonary fibrosis, Gli1^+^ MSCs inhibit the activation of BMP in airway progenitor cells’ microenvironment by upregulating the hedgehog(Hh) signal. During fiber repair, the proximal bronchial/airway epithelium can appear ectopic in the distal lung, which is characterized by metaplasia of KRT5^+^ basal cells arranged on the alveoli along the fibrous scar to form an air containing cyst [97, 98]. The presence of metaplastic KRT5^+^ cells is associated with increased disease severity and reduced survival [99].

Under physiological conditions, KRT5^+^ basal cells exist in the trachea and large airway of mouse lung, and SFTPC^+^ type 2 cells exist in the distal alveolar sac to produce functional alveolar epithelium. The upregulation of Hh signal inhibits BMP signal in microenvironment, which makes KRT5^+^ airway progenitor cells have metaplasia and differentiation, and promotes the differentiation of adaptive alveoli into SFTPC^+^ epithelium [97].

## Conclusion and discussion

Mesenchymal stem cells and microenvironment have distinct characteristics, but they are geographically and functionally linked. MSCs affect their microenvironment through different ways, such as immunomodulation, hematopoietic support and tissue regeneration. The microenvironment also regulate the differentiation, proliferation and function of MSCs. In the pathologic microenvironment, MSCs may aggravate or alleviate the disease. Controversy still exists in the application of MSCs, because the same microenvironment may cause contrary effects on MSCs [100]. Many clinical trials about MSCs therapy have been conducted, but its questionable safety and efficacy continue to limit its application [43]. Moreover, changing the microenvironment may cause good or bad influences on MSCs therapy and disease progression. The microenvironment is a complex entity, so it can be difficult for the study and application. With further research of the changes in their functions, composition and location in the related pathological process, the mechanism of disease occurrence and development will be clearer, and more innovative treatments will be created.

## Material and methods

The review analyzed the results of studies on MSCs and their microenviroment that were mainly found in PubMed and Web of Science. Search terms and phrases such as “MSCs microenvironment”, “MSCs immunomodulation”, “microenvironment affect MSCs differentiation”, “interaction between MSC and microenvironment”, “tumor microenvironment”, “hypoxia microenvironment”, “bone marrow microenvironment”, and “MSCs tissue repair” were used to identify articles that could help researchers explore the topic. The search include state-of-the-art articles that were published in English in the two electronic databases. The abstracts of the available articles were carefully reviewed to determine their quality and appropriateness, and the aim, research design, results, and conclusions in each of the selected articles were examined.

Inclusion criteria: originality,innovation,and reliability.

Exclusion criteria: out of date and irrelevant articles.

One hundred seventy-four studies were screened, and 101 studies were included in the review.

## Data Availability

Please contact the corresponding author for data requests.

## Abbreviations

MSCs: Mesenchymal stem cells
PGE2: Prostaglandin E2
IDO: Indoleamine 2,3-dioxygenase
Treg cells: Regulatory T cells
TGF-β: Transforming growth factor beta
IFN-γ: Interferon-γ
RORγt: Orphan nuclear receptor gamma
Foxp3: Forkhead box P3
CIA: Collagen-induced arthritis
LIF: Leukemia inhibitory factor
DC: Dendritic cells
IL-10: Interleukin-10
HSCs: Hematopoietic stem cells
HGF: Hepatocyte growth factor
IGF-1: Insulin-like growth factor
EGF: Epithelial growth factor
NGF: Nerve growth factor
TGFA: Transforming growth factor-α
VEGF: Vascular endothelial growth factor
AD-MSCs: Adipose derived MSCs
TDSCs: Tendon mesenchymal stem cells
ERK1/2: Extracellular signal-regulated protein kinases 1 and 2
FAK: Focal adhesion kinase
IVD: Intervertebral disc
Hh: Hedgehog
BMP: Basic metabolic panel
TSG6: Tumor necrosis factor (TNF)-stimulated gene 6 protein
SASP: Senescence-associated secretory phenotype
PDGF: Platelet-derived growth factor
bFGF: Basic fibroblast-like growth factor
GDF-5: Growth differentiation factor-5
